# Supplementary material for: Screening of Phytochemical, Antimicrobial, and Antioxidant Properties of Juncus acutus from Northeastern Morocco
Source: Life (Basel). 2023 Oct 29;13(11):2135. doi: 10.3390/life13112135 (PMC10672446; doi:10.3390/life13112135)
Supplement: Supplementary file 1 [file life-13-02135-s001.zip › life-2603800-supplementary.pdf]

life

Supplementary file

Screening of Phytochemical, Antimicrobial, and Antioxidant Properties of *Juncus acutus* from Northeastern Morocco

Yousra Hammouti [1], Amine Elbouzidi [1], Mohamed Taibi [1,2], Reda Bellaouchi [3], El Hassania Loukili [2], Mohamed Bouhrim [4,5], Omar M. Noman [6], Ramzi A. Mothana [6], Mansour N. Ibrahim [7], Abdeslam Asehraou [3], Bouchra El Guerrouj [1,2], and Mohamed Addi [1,*]

[1] Laboratoire d'Amélioration des Productions Agricoles, Biotechnologie et Environnement (LAPABE), Faculté des Sciences, Université Mohammed Premier, Oujda 60000, Morocco
[2] Centre de l'Oriental des Sciences et Technologies de l'Eau et de l'Environnement (COSTEE), Université Mohammed Premier, Oujda 60000, Morocco
[3] Laboratory of Bioresources, Biotechnology, Ethnopharmacology and Health, Faculty of Sciences, Mohammed First University, Boulevard Mohamed VI, B.P. 717, Oujda 60000, Morocco
[4] Laboratories TBC, Laboratory of Pharmacology, Pharmacokinetics and Clinical Pharmacy, Faculty of Pharmacy, University of Lille, 59000 Lille, France
[5] Laboratory of Biological Engineering, Team of Functional and Pathological Biology, Faculty of Sciences and Technology, University Sultan Moulay Slimane, Beni Mellal 23000, Morocco
[6] Department of Pharmacognosy, College of Pharmacy, King Saud University, Riyadh 11451, Saudi Arabia
[7] Department of Agricultural Engineering, College of Food and Agriculture Sciences, King Saud University, Riyadh 11451, Saudi Arabia
* Correspondence: m.addi@ump.ac.ma

Table S1. Physiochemical and drug-likeness analysis of the major compounds found in *J. acutus* Leaves extract (JALE), and *J. acutus* roots extract (JARE). (1) Gallic acid, (2) Caffeic acid, (3) Ferulic acid, (4) Catechin, (5) Syringic acid, (6) Catechin hydrate, (7) 4-Hydroxybenzoic acid, (8) Naringin, (9) Cinnamic acid, (10) p-coumaric acid, (11) Sinapic acid, (12) Rutin hydrate, (13) Succinic acid, (14) Quercetin 3-O-β-D-glucoside, (15) Rutin, (16) Quercetin, (17) Kaempferol.

**N°**	**Presence**		**HBD**	**HBA**	**TPSA ($Å^2$)**	**Log Po/w (WLOGP)**	**Log S (SILICO S-IT)**	**Lipinski's Rule of Five**	**Veber filter**
	**JALE**	**JARE**							
**1**	+	+	4	5	97.99	0.50	-0.20 (+++)	Yes; 0 violation	Yes; 0 violation
**2**	+	-	3	4	77.76	1.09	0.75 (+++)	Yes; 0 violation	Yes; 0 violation
**3**	++	-	2	4	66.76	1.39	1.26 (+++)	Yes; 0 violation	Yes; 0 violation
**4**	+	-	5	6	110.38	1.22	-2.14 (+++)	Yes; 0 violation	Yes; 0 violation
**5**	+	+	2	3	57.53	1.09	-1.17 (+++)	Yes; 0 violation	Yes; 0 violation
**6**	+	+	6	7	119.61	1.16	0.98 (+++)	Yes; 1 violation: NHorOH>5	Yes; 0 violation
**7**	+	+	2	3	57.53	1.09	-1.17 (+++)	Yes; 0 violation	Yes; 0 violation
**8**	+	+	8	14	225.06	-1.49	-0.49 (+++)	No; 3 violations: MW>500, NorO>10, NHorOH>5	No; 1 violation: TPSA> 140
**9**	+	+	1	2	37.30	1.68	1.70 (+++)	Yes; 0 violation	Yes; 0 violation
**10**	+	+	2	3	57.53	1.38	1.22 (+++)	Yes; 0 violation	Yes; 0 violation
**11**	-	+	2	5	75.99	1.40	1.33 (+++)	Yes; 0 violation	Yes; 0 violation
**12**	+	+	11	17	278.66	-1.75	-2.11 (+++)	No; 3 violations: MW>500, NorO>10, NHorOH>5	No; 1 violation: TPSA>140
**13**	-	+	2	4	74.60	-0.06	0.61 (+++)	Yes; 0 violation	Yes; 0 violation
**14**	-	+	8	12	210.51	-0.54	-0.59 (+++)	No; 2 violations: NorO>10, NHorOH>5	No; 1 violation: TPSA>140
**15**	+	+	10	16	269.43	-1.69	-2.11 (+++)	No; 3 violations: MW>500, NorO>10, NHorOH>5	No; 1 violation: TPSA>140
**16**	-	+	5	7	131.36	1.99	-3.24 (+++)	Yes; 0 violation	Yes; 0 violation
**17**	-	+	4	6	111.13	2.28	2.03 (+++)	Yes; 0 violation	Yes; 0 violation

HBD: Donors of Hydrogen Bonds; HBA: Acceptors of Hydrogen Bonds; Log Po/w: Partition Coefficient P; Log S: Solubility; (+++) High Solubility+: present; -:absent

Table S2. The (ADME) pharmacokinetic characteristics of the identified compounds present JALE, and JARE. (1) Gallic acid, (2) Caffeic acid, (3) Ferulic acid, (4) Catechin, (5) Syringic acid, (6) Catechin hydrate, (7) 4-Hydroxybenzoic acid, (8) Naringin, (9) Cinnamic acid, (10) p-coumaric acid, (11) Sinapic acid, (12) Rutin hydrate, (13) Succinic acid, (14) Quercetin 3-O-β-D-glucoside, (15) Rutin, (16) Quercetin, (17) Kaempferol.

Prediction	1	2	3	4	5	6	7	8	9	10	11	12	13	14	15	16	17
**ADME Prediction**																	
**Absorption Parameters**																	
**Bioavailability score**	0.56	0.56	0.85	0.55	0.85	0.55	0.85	0.17	0.85	0.85	0.56	0.17	0.85	0.85	0.17	0.55	0.55
**Caco-2 Permeability**	-0.081	0.634	1.151	-0.283	0.176	0.159	1.151	-0.658	1.717	1.158	0.136	-1.248	0.603	0.242	-0.949	-0.229	0.032
**Intestinal Absorption (%)**	43.37	69.40	83.96	68.82	93.68	63.91	83.96	25.79	94.83	91.11	93.89	25.74	71.74	47.99	23.44	77.20	74.29
**Distribution Parameters**																	
**Log K_p (cm/s)**	-2.735	-2.722	-2.723	-2.735	-2.72	-2.735	-2.723	-2.735	-2.695	-2.323	-2.72	-2.735	-2.735	-2.735	-2.735	-2.735	-2.735
**VDss**	-1.855	-1.098	-1.557	1.027	-1.367	1.023	-1.557	0.619	-1.051	-0.406	-0.797	0.576	-1.013	1.846	1.663	1.559	1.274
**BBB Permeability**	-1.102	-0.647	-0.334	-1.054	-0.239	-1.06	-0.334	-1.600	0.446	-0.257	-0.198	-2.187	-0.163	-1.688	-1.899	-1.098	-0.939
**Metabolism Parameters**																	
**CYP2D6, and CYP3A4 Substrate**	No	No	No	No	No	No	No	No	No	No	No	No	No	No	No	No	No
**CYP2D6, and CYP3A4 Inhibitors**	No	No	No	No	No	No	No	No	No	No	No	No	No	No	No	No	No
**Excretion Parameters**																	
**Total Clearance**	0.518	0.508	0.593	0.183	0.623	0.181	0.593	0.318	0.781	0.672	0.741	-0.081	0.722	0.394	-0.369	0.407	0.477
**Renal OCT2 Substrate**	No	No	No	No	No	No	No	No	No	No	No	No	No	No	No	No	No

BBB: blood-brain barrier; Log BB > 0.3, molecule BBB permeant, Log BB < -1 molecule poorly distributed across the BBB.

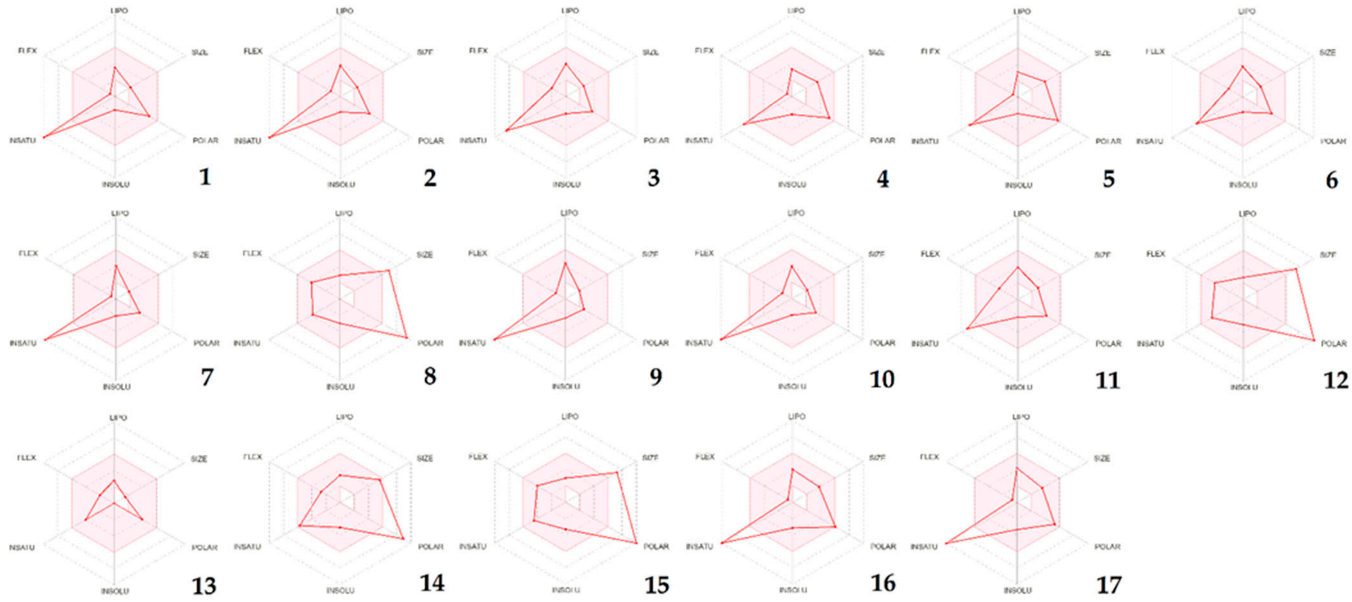

Figure S1. JALE, and JARE compounds' bioavailability radars based on six physicochemical properties (lipophilicity, size, polarity, solubility, flexibility, and saturation). Note: (1) Gallic acid, (2) Caffeic acid, (3), Ferulic acid (4), Catechin, (5) Syringic acid, (6) Catechin hydrate, (7) 4-Hydroxybenzoic acid, (8) Naringin, (9) Cinnamic acid, (10) p-coumaric acid, (11) Sinapic acid, (12) Rutin hydrate, (13) Succinic acid, (14) Quercetin 3-O-β-D-glucoside, (15) Rutin, (16) Quercetin, (17) Kaempferol.

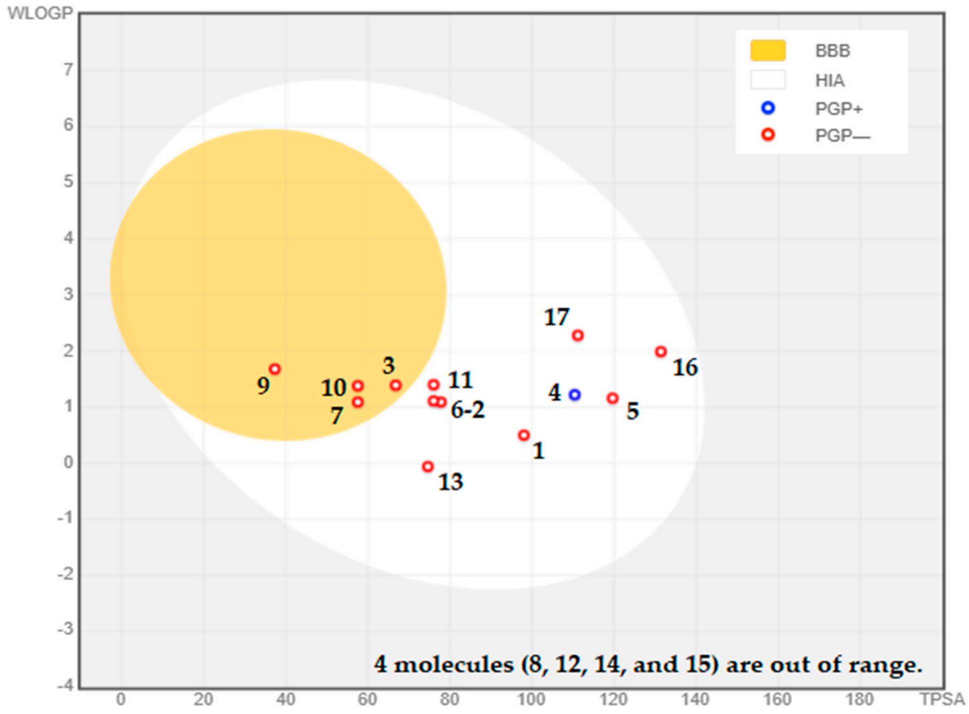

Figure S2. The BOILED-EGG Framework: Employed for Evaluating JALE, and JARE Composition Regarding Blood-Brain Barrier Permeability, Gastrointestinal Absorption, and Interaction with P-glycoprotein as Substrates or Inhibitors. (1) Gallic acid, (2) Caffeic acid, (3), Ferulic acid (4), Catechin, (5) Syringic acid, (6) Catechin hydrate, (7) 4-Hydroxybenzoic acid, (8) Naringin, (9) Cinnamic acid, (10) p-coumaric acid, (11) Sinapic acid, (12) Rutin hydrate, (13) Succinic acid, (14) Quercetin 3-O-β-D-glucoside, (15) Rutin, (16) Quercetin, (17) Kaempferol.

Table S3. Prediction of toxicity, and the toxic endpoints of the major compounds found in JALE, and JARE. (1) Gallic acid, (2) Caffeic acid, (3) Ferulic acid, (4) Catechin, (5) Syringic acid, (6) Catechin hy drate, (7) 4-Hydroxybenzoic acid, (8) Naringin, (9) Cinnamic acid, (10) p-coumaric acid, (11) Sinapic acid, (12) Rutin hydrate, (13) Succinic acid, (14) Quercetin 3-O-β-D-glucoside, (15) Rutin, (16) Quercetin, (17) Kaempferol.

N	Predicted LD_{50} (mg/kg)	Class	Hepatotoxicity		Cytotoxicity		Immunotoxicity		Mutagenicity		Carcinogenicity	
			Predi.	Prob.	Predi.	Prob.	Predi.	Prob.	Predi.	Prob.	Predi.	Prob.
**1**	2000	IV	Ina.	0.94	Ina.	0.91	Ina.	0.99	Ina.	0.61	**Act.**	**0.56**
**2**	2980	V	Ina.	0.98	Ina.	0.86	Ina.	0.50	Ina.	0.57	**Act.**	**0.78**
**3**	1772	IV	Ina.	0.96	Ina.	0.88	**Act.**	**0.91**	Ina.	0.51	Ina.	0.61
**4**	10000	VI	Ina.	0.55	Ina.	0.84	Ina.	0.96	Ina.	0.72	Ina.	0.51
**5**	10000	VI	**Act.**	**0.55**	Ina.	0.84	Ina.	0.97	Ina.	0.73	Ina.	0.76
**6**	1700	IV	Ina.	0.93	Ina.	0.97	Ina.	0.97	Ina.	0.58	Ina.	0.70
**7**	2200	V	Ina.	0.99	Ina.	0.86	Ina.	0.99	Ina.	0.52	Ina.	0.51
**8**	2300	V	Ina.	0.73	Ina.	0.66	**Act.**	**0.99**	Ina.	0.81	Ina.	0.90
**9**	2500	V	Ina.	0.96	Ina.	0.83	Ina.	0.95	**Act.**	**0.54**	Ina.	0.82
**10**	1048	IV	Ina.	0.99	Ina.	0.98	Ina.	0.78	Ina.	0.68	Ina.	0.64
**11**	1772	IV	Ina.	0.87	Ina.	0.96	**Act.**	**0.89**	Ina.	0.54	Ina.	0.67
**12**	2850	V	Ina.	0.92	Ina.	0.81	Ina.	0.86	**Act.**	**0.53**	**Act.**	**0.51**
**13**	2260	V	Ina.	0.98	Ina.	0.75	Ina.	0.99	Ina.	0.83	Ina.	0.80
**14**	1034	IV	Ina.	0.98	Ina.	0.86	Ina.	0.99	**Act.**	**0.51**	Ina.	0.67
**15**	5000	V	Ina.	0.88	Ina.	0.64	**Act.**	**0.98**	Ina.	0.80	Ina.	0.91
**16**	159	III	Ina.	0.51	Ina.	0.99	Ina.	0.87	Ina.	0.69	**Act.**	**0.68**
**17**	3919	V	Ina.	0.52	Ina.	0.98	Ina.	0.96	Ina.	0.68	Ina.	0.72
